# What is an “ongoing” clinical trial? An analysis of different sources revealed heterogeneous definitions of when a clinical trial starts and ends: a meta-research study

**DOI:** 10.1590/1516-3180.2022.0634.R2.100523

**Published:** 2023-07-17

**Authors:** Rafael Leite Pacheco, Ana Luiza Cabrera Martimbianco, Rachel Riera

**Affiliations:** I MSc. PhD. Physician and Researcher, Centre of Health Technology Assessment, Hospital Sírio-Libanês, São Paulo (SP), Brazil. Researcher, Universidade Federal de São Paulo (UNIFESP), São Paulo (SP), Brazil. Professor, Centro Universitário São Camilo, São Paulo (SP), Brazil.; Universidade Federal de São Paulo, São Paulo, SP, Brazil; Centro Universitário São Camilo, São Paulo, SP, Brazil; II MSc, PhD. Physiotherapist and Researcher, Centre of Health Technology Assessment, Hospital Sírio-Libanês, São Paulo (SP), Brazil. Professor, Universidade Metropolitana de Santos (UNIMES), Santos (SP), Brazil.; Universidade Metropolitana de Santos, Santos, SP, Brazil; III MSc. PhD. Physician Adjunct Professor, Discipline of Evidence-Based Health, Escola Paulista de Medicina (EPM), Universidade Federal de São Paulo (UNIFESP), São Paulo (SP), Brazil. Coordinator, Centre of Health Technology Assessment, Hospital Sírio-Libanês, São Paulo (SP), Brazil.; Hospital Sírio-Libanês, Centre of Health Technology Assessment, São Paulo, SP, Brazil

**Keywords:** Randomized controlled trials as topic, Controlled clinical trials as topic, Clinical trial [publication type], Study classification, Study category, Ongoing studies

## Abstract

**BACKGROUND:**

Although the concept of an “ongoing study” seems self-explanatory, it is difficult to determine whether a trial is underway.

**OBJECTIVE:**

To analyze the definitions of “ongoing clinical trial” across different clinical trial registries, methodological guidelines, and other sources.

**DESIGN AND SETTING:**

This meta-research study was conducted at the Universidade Federal de São Paulo (UNIFESP), Brazil.

**METHODS:**

We performed a cross-sectional analysis of relevant clinical trial registry databases, methodological guidelines for conducting systematic reviews, and other sources that would define or regulate clinical trials.

**RESULTS:**

We identified various heterogeneous definitions used by eligible sources at both the start and end of a clinical trial. The starting criteria used were as follows: when the team is planning the protocol, when permission is given to conduct the study, or when the first participant is enrolled. Some sources used the time at which the last outcome data was collected as a criterion to determine the end of the trial. The International Committee of Medical Journal Editors stated that a study is still “ongoing” during the analysis process. Several sources use a vague definition or present no clear criteria for defining the start or end of a study.

**CONCLUSION:**

The concept of “ongoing clinical trials” lacks a transparent and homogeneous definition across relevant sources. A consensus on this concept is important to facilitate the evaluation of available evidence and conduct research synthesis. Further efforts are necessary to determine the best definition for the start and end of a clinical trial.

## INTRODUCTION

The selection and classification of primary studies is an elementary and key component when assessing medical literature or conducting a systematic review.^1–4^ However, these processes can be highly subjective and lead to different decisions or judgments.^5^

A common approach, mainly in interventions/effectiveness reviews, is to classify any study that has not been completed as “ongoing clinical trial.”^4^ The problem with this approach is that the definition of when a study starts or ends may not be uniform and impose challenges when assigning a trial as “ongoing.”^4^

Although the concept of an ongoing study appears self-explanatory, it is difficult to determine whether a trial is underway.

For instance, one may state that a clinical trial begins when the first patient is enrolled or has received the first intervention dose or even when the first outcome data is collected. The start of a study can also be defined as the time when the primary objective is conceived or when the team is assembled. A study’s end may also have different definitions, such as when the last participant was enrolled, when the last outcome data were collected, or when all analyses were completed.

Considering the challenges in uniformly categorizing ongoing studies, we aimed to map the definition criteria adopted by major sources.

## OBJECTIVE

This study aimed to analyze the definitions of an “ongoing clinical trial” across different clinical trial registries and methodological guidelines.

## METHODS

We conducted a meta-research study at the Núcleo de Ensino e Pesquisa em Saúde Baseada em Evidências e Avaliação Tecnológica em Saúde from the Universidade Federal de São Paulo, Brazil.

We performed a cross-sectional analysis in the following sources to investigate their assumptions adopted to define the start and end of a clinical trial:

- National Institutes of Health - Clinicaltrials.gov/^6^
- International Standards for Clinical Trial Registration (ISRCTN) registry^7^
- European Union Clinical Trials register^8^
- World Health Organization - International Clinical Trials Registry Platform^9^
- Campbell Collaboration Information Retrieval Guide^10^
- Cochrane Handbook for Systematic Reviews of Interventions^4^
- Joanna Briggs Institute Manual for Evidence Synthesis^11^
- International Committee of Medical Journal Editors^12^

We also extracted their definitions to detect any mention regarding “ongoing clinical trials.”

The sources were chosen based on our expertise, and we attempted to cover information from any relevant clinical trial registry database, methodological guidelines to conduct systematic reviews, and other sources that would define or regulate clinical trials.

The search and data extraction date was January 16, 2022. Data extraction was aided using an Excel sheet and the results were presented narratively.

## RESULTS

After analyzing eligible sources, we found various definitions for both the start and end of a clinical trial (Table 1).

**Table 1:** Definitions of “ongoing” trials by different sources

Source	Definition			Comments
	Study start	Study end or completion	Additional relevant information regarding definition of “ongoing” studies	
	Clinical trial registry database			
NIH Clinicaltrials.gov/^6^	“Study start date: The actual date on which the first participant was enrolled in a clinical study.”	“Study completion date: The date on which the last participant in a clinical study was examined or received an intervention/treatment to collect final data for the primary outcome measures, secondary outcome measures, and adverse events (that is, the last participant’s last visit).”	“Active, not recruiting: The study is ongoing, and participants are receiving an intervention or being examined, but potential participants are not currently being recruited or enrolled.”	This source utilizes several categories for studies status. Considering the information provided, it is assumed that an ongoing trial is one that is still recruiting and/or collecting outcome data.
ISRCTN registry^7^	“A study starts when you begin planning the design of the study and developing the protocol.”	“The end date of your study should be stated in your study protocol. In many cases, it is expected to be the last date that data is collected.”	NF	This source considers a study as “ongoing” from the planning of the design to the last data collection.
EU Clinical Trials register^8^	“Start Date: The date on which the clinical trial commenced.” In the registry’s presentation, the field “start date” is marked, indicating that the start date is the date when the study received “authorization to proceed.”	“Date of the global end of the trial: This is the date on which the trial is ended in all countries.”	NF	This source defines the study start date as the time where the study was authorized and give no clear definition of the study end date.
WHO - International Clinical Trials Registry Platform^9^	NF	“Date of study completion: The date on which the final data for a clinical study were collected (commonly referred to as, ‘last subject, last visit’).”	NF	This source defines the study completion as the end of outcome data collection. No clear definition was given as to when the study starts; it only presented the “date of study registration” and “date of first enrollment.”
**Methodological guidance to conduct systematic reviews**				
Campbell Collaboration Information Retrieval Guide^10^	NF	NF	NF	Although authors highlight the importance of considering “ongoing studies,” there is no clear definition to when a study starts or ends.
Cochrane Handbook for Systematic Reviews of Interventions^4^	NF	NF	“Users of the review will be interested to learn of any potentially relevant studies that have not been completed.”	This source highlights the importance of considering ongoing studies and makes a clear distinction between “unpublished” and “ongoing studies.” However, there is no clear definition to when a study starts or ends.
Joanna Briggs Institute Manual for Evidence Synthesis^11^	NF	NF	NF	No clear definition was mentioned.
**Other sources**				
International Committee of Medical Journal Editors^12^	NF	NF	“The ICMJE considers trials that began enrollment before July 1, 2005 to be ‘ongoing’ if the investigators were still collecting, cleaning, or analyzing data as of July 1, 2005.”	This source defines the end of a trial after final data analysis.

EU = European Union; ICMJE = International Committee of Medical Journal Editors; ISRCTN = International Standards for Clinical Trial Registration; NIH = National Institutes of Health; NF = not found; WHO = World Health Organization.

The ISRCTN registry considers a study to start when the team is planning the protocol,^7^ and the European Union Clinical Trials register defines the study start date as when permission is given to conduct the study.^8^ The National Institutes of Health (Clinicaltrials.gov/) registry defines study initiation as when the first participant is enrolled.^6^

Some sources used the time at which the last outcome data was collected as a criterion to determine the end of the trial. The International Committee of Medical Journal Editors states that a study is still “ongoing” during the analysis process,^12^ which means that the study ends only after the end of the data collection process.

Several sources use a vague definition or present no clear criteria for defining the start or end of a study. Methodological guidelines for conducting systematic reviews, such as the *Cochrane Handbook for Systematic Reviews of Interventions* ^4^ and *Campbell Collaboration Information Retrieval Guide*,^10^ often highlight the importance of considering the ongoing status of clinical trials as an inclusion criterion. However, they still do not provide a clear definition of what “ongoing” means.

## DISCUSSION

Any evaluation of the literature will be affected by the transparency of the study reports. It is difficult to assess the pool of available evidence and evidence projected to be available in the near future without a homogeneous definition of “ongoing” studies.

Another problem is the development of research synthesis. The selection and classification of primary studies is an elementary and key component when conducting a systematic review of the literature. However, these processes can be highly subjective and can lead to divergent decisions or judgments among reviewers.

In light of these definitions of “ongoing studies,” the process of categorizing studies may vary substantially among reviewers, thereby affecting reproducibility and transparency of the reviews.

The category of ongoing clinical trials is not the only one that lacks consensus regarding its definition. In a previous study,^5^ we analyzed the justifications for considering a study to be “awaiting classification” in a sample of published systematic reviews, and we found a high proportion of conflicting or unclear justifications.

Our results showed that study categorization still lacks clear definitions and recommendations and that strict criteria need to be applied to increase transparency and improve the reproducibility of systematic reviews. A homogeneous and clear definition of ongoing studies must be adopted through trial registration databases and systematic review development guidelines.

Our analysis has limitations because we adopted an unstructured search for relevant sources. However, although anecdotal, our results showed that major sources have heterogeneous definitions for the start and end of a clinical trial, and it is reasonable to assume that this is a widespread problem in the medical literature.

## CONCLUSION

The concept of “ongoing” clinical trial lacks transparent and homogeneous definitions across relevant sources. A consensus on this concept is important to facilitate the evaluation of available evidence and conduct research synthesis and systematic reviews. Further efforts are necessary to determine the best definition for the start and end of a clinical trial.

## References

[1] Shea BJ, Reeves BC, Wells G (2017). AMSTAR 2: a critical appraisal tool for systematic reviews that
include randomised or non-randomised studies of healthcare interventions, or
both. BMJ.

[2] Page MJ, McKenzie JE, Bossuyt PM (2021). The PRISMA 2020 statement: an updated guideline for reporting
systematic reviews. BMJ.

[3] Page MJ, Moher D, Bossuyt PM (2021). PRISMA 2020 explanation and elaboration: updated guidance and
exemplars for reporting systematic reviews. BMJ.

[4] Higgins JPT, Thomas J, Chandler J (2019). Cochrane Handbook for Systematic Reviews of Interventions version 6.0
(updated July 2019).

[5] Pacheco RL, Latorraca COC, Cabrera Martimbianco AL, Riera R (2020). Reasons for “awaiting classification” studies are often
inadequate and underreported: a cross-sectional analysis of cochrane
reviews. J Clin Epidemiol.

[6] National Institutes of Health – Clinical trials.gov. Glossary of Common Site Terms.

[7] International Standards for Clinical Trial Registration Definitions.

[8] European Clinical Trials Register Glossary of Terms used in EU Clinical Trials Register.

[9] World Health Organization. WHO Data Set WHO Trial Registration Data Set (Version 1.3.1).

[10] Kugley S, Wade A, Thomas J (2017). Searching for studies: a guide to information retrieval for
Campbell systematic reviews. Campbell Systematic Reviews.

[11] Tufanaru C, Munn Z, Aromataris E, Campbell J, Hopp L, Aromataris E, Munn Z (2020). JBI Manual for Evidence Synthesis.

[12] International Committee of Medical Journal Editors (2014). Clinical Trials Registration.

